# Thermal and Physico-Mechanical Characterizations of Thromboresistant Polyurethane Films

**DOI:** 10.3390/bioengineering6030069

**Published:** 2019-08-14

**Authors:** Aaron C. Wilson, Shih-Feng Chou, Roberto Lozano, Jonathan Y. Chen, Pierre F. Neuenschwander

**Affiliations:** 1 Department of Mechanical Engineering, College of Engineering, The University of Texas at Tyler, 3900 University Blvd, Tyler, TX 75799, USA; 2 School of Human Ecology, College of Natural Sciences, The University of Texas at Austin, Austin, TX 78712, USA; 3 Department of Cellular and Molecular Biology, The University of Texas Health Science Center at Tyler, Tyler, TX 75708, USA

**Keywords:** polyurethane, thermal properties, mechanical properties, thrombosis, blood clotting

## Abstract

Hemocompatibility remains a challenge for injectable and/or implantable medical devices, and thromboresistant coatings appear to be one of the most attractive methods to down-regulate the unwanted enzymatic reactions that promote the formation of blood clots. Among all polymeric materials, polyurethanes (PUs) are a class of biomaterials with excellent biocompatibility and bioinertness that are suitable for the use of thromboresistant coatings. In this work, we investigated the thermal and physico-mechanical behaviors of ester-based and ether-based PU films for potential uses in thromboresistant coatings. Our results show that poly(ester urethane) and poly(ether urethane) films exhibited characteristic peaks corresponding to their molecular configurations. Thermal characterizations suggest a two-step decomposition process for the poly(ether urethane) films. Physico-mechanical characterizations show that the surfaces of the PU films were hydrophobic with minimal weight changes in physiological conditions over 14 days. All PU films exhibited high tensile strength and large elongation to failure, attributed to their semi-crystalline structure. Finally, the in vitro clotting assays confirmed their thromboresistance with approximately 1000-fold increase in contact time with human blood plasma as compared to the glass control. Our work correlates the structure-property relationships of PU films with their excellent thromboresistant ability.

## 1. Introduction

One of the most attractive engineering approaches toward the treatment of many diseases and disabilities is the use of injectable and/or implantable medical devices [1]. For example, vascular stents are placed in the blood vessels to provide sufficient blood flow. Vascular stents provide structural support to the regrowth of the endothelial cells during recovery, while allowing the integration with other important biomedical cues, such as drug release ability. Since vascular stents are difficult to remove over time, it is ideal to ensure that the stents are utilized to the fullest extent of their potential by reducing the inflammatory response produced by the body’s immune system. One of the most widely used methods to solve this issue has been the use of thromboresistant coatings on the surface of the stents to minimize the risk associated with the body’s rejection of the device [2]. 

The first step towards determining the effectiveness of a thromboresistant coating is to understand how the body will react to the introduction of foreign materials into the bloodstream, such as the polyurethane coatings [3]. In a healthy body, the walls of the blood vessels throughout the circulatory system, which are directly in contact with the blood, consist of arrays of vascular endothelial cells. These endothelial cells are important for a number of functions when healthy, including the prevention of unwanted coagulation. When damaged, they become thrombotic and the coagulation cascade is initiated [4]. Of the two ways by which the coagulation process is initiated, the intrinsic contact pathway is the main focus for this application, as it is activated when foreign materials are introduced into the bloodstream. This contact activates the plasma protein called factor XII, along with a mass activation (or cascade) of other enzymes and proteins, which triggers the activation of factor IXa [5]. The cascade concludes with the polymerization of the protein fibrin and the activation of platelets, which together form blood clots [6].

Polyurethanes (PUs) are widely used in many biomedical applications due to their excellent biocompatibility [7,8] and slow degradability [9,10,11]. They are made by a variety of processes with structural components containing alternating soft segments (polyols) and hard segments (diisocyanates) at specific stoichiometric ratios, depending on the desired properties of the PUs [12]. Each segment has a unique functional group, and urethane linkages (–NHCO–) are formed when the hydroxyl groups (OH) in the soft segments and the isocyanate groups (R-NCO) in the hard segments are combined together [13].

PUs are categorized as elastomers with high tensile strength, large elasticity, as well as low crystallinity [14]. These properties, along with their excellent biocompatibility, have led to widespread research into their viability in biomedical applications. PU films have proven to be well suited for numerous medical-grade applications, including the use of protective coating on implantable devices [15]. Another example of PUs is the use as drug-release carriers, which either degrade to release drugs into a patient or have the drugs attached externally so that they will directly degrade over time [16]. Similarly, PU films have displayed excellent thromboresistant ability, which makes them very useful for coatings on implantable devices to prolong their lifespan in the body [17]. Others have synthesized PUs to create porous scaffolds that can be used to repair or replace damaged organs or tissues [18]. 

In this study, four PU films, two ester-based and two ether-based, were studied on their potential use in thromboresistant coatings for medical implants. Since the samples were kindly supplied by a manufacturer, we first determined their physical appearance of the films. Later, the chemical structure of the PU films was investigated by Fourier-transform infrared (FTIR) spectroscopy. In addition, we evaluated their thermal properties by using differential scanning calorimetry (DSC) and thermal gravimetric analysis (TGA), as well as their crystallinity by using X-ray diffraction (XRD) technique. The physico-mechanical properties of the PU films were then examined by water contact angle and water absorption/degradation study, as well as tensile testing. Lastly, we performed in vitro activated partial thrombin time (APTT) assays using human blood plasma on the various PU films to determine the clotting time through contact pathway. We show that the structure-property relationships of the investigated PU films, including thermoplastic behaviors, low crystallinity, hydrophobicity, slow degradability, excellent mechanical properties, and thromboresistant ability, are suitable for medical coatings of implantable devices.

## 2. Materials and Methods

### 2.1. Materials

Four types of polyurethane (PU) films with an average shore hardness 85A and 92A were purchased from Jiaxing Inch Eco Materials Co., Ltd. (Zhejiang, China). In addition, two types of PU films contained ester-based copolymer, whereas the other two types of PU films contained ether-based copolymer. The thickness of the PU films ranged from 60 to 200 μm, measured by a digital thickness gauge (resolution = 10 μm). Table 1 details the specifications of the four PU films used in this study.

Citrated Pooled Normal Human Plasma (1.0 mL) was bought from George King Bio-Medical, Inc. (Overland Park, KS, USA). Calcium chloride from Sigma-Aldrich (St. Louis, MO, USA) was prepared in deionized water at a working concentration of 25 mM. All the other chemicals were of reagent grade and used as received without further purification.

### 2.2. Fourier-Transform Infrared (FTIR) and Nuclear Magnetic Resonance (NMR) Spectroscopy

The chemical structure of various PU samples was measured using a Thermo Fisher Scientific Nicolet iS10 ATR-FTIR spectrophotometer (Walthem, MA, USA), according to the previously published method [19]. The spectra were recorded between 4000 and 650 cm^−1^ with a resolution of 8 cm^−1^. Peak information from various PU sample spectra were analyzed using OMNIC^TM^ software.

The ^1^H spectra of various PU samples were recorded on an Agilent NMR system at 400 MHz. PU samples were dissolved in dimethyl sulfoxide-d6 at 3 wt%, and DSS (3-(trimethylsilyl)-propanesulfonic acid, sodium salt) was used as the internal reference for the ^1^H chemical shifts.

### 2.3. Differential Scanning Calorimetry

A Shimadzu DSC-50 (Kyoto, Japan) was used to analyze the crystallinity, glass transition, and melting temperatures of film samples at a scanning rate of 20 °C/min between 25 and 200 °C. The weight of the specimens ranged between 6 and 10 mg. Results were averaged on three independent measurements.

### 2.4. Thermal Gravimetric Analysis

Mass changes of samples during thermal decomposition were investigated by thermal gravimetric analysis using a Shimadzu TGA-50 Thermal Analyzer (Kyoto, Japan). The heating rate was 20 °C/min from 10 to 650 °C under N_2_ gas with a flow rate of 30%. The weight of tested sample ranged between 15 and 18 mg. Results were averaged on three independent measurements.

### 2.5. X-ray Diffraction

The diffraction patterns of various PU films were recorded using Rigaku R-Axis Spider^TM^ (Tokyo, Japan). X-ray source was generated from copper Kα wavelength (λ = 1.541 Å) at a voltage of 40 kV and a 40 mA current. The diffractometer was used in reflection mode. The XRD data was collected at a resolution of 2θ = 0.05°, from 2θ = 4° to 40°.

### 2.6. Water Contact Angle and Water Absorption

The surface wettability of various PU films was quantified by water contact angle measurements. A single droplet of phosphate-buffered saline (4 μL) was placed on the surface of the films, and the contact angle was measured using ImageJ (National Institutes of Health, Bethesda, MD, USA). Results were averaged on three independent measurements.

Water absorption data were obtained through immersing disk PU specimens (1" in diameter) into 3 mL of phosphate-buffered saline for up to 2 weeks. Samples were kept in an incubator at 37 °C under constant shaking. At pre-determined time points, PU samples were removed from the liquid and gently wiped-dry to record the mass. Percentage weight change in water content was calculated using the equation below.

$$\%Weight\;Change=\;\frac{W_{t}-W_{i}}{W_{i}}\;\times \;100\%$$

where *W_i_* is the initial dry weight and *W_t_* is the dry weight at each time interval. Results were averaged on three independent measurements.

### 2.7. Mechanical Testing

An Instron 3340 universal mechanical tester (Norwood, MA, USA) was used for the tensile testing of the PU films according to ASTM standard D882-18 (e.g., 23 ± 2 °C and 50 ± 5% RH) [20]. PU films were cut using a dog-bone die according to ASTM standard D1708-96 (22 mm in nominal length and 5 mm in width) [21]. Tensile tests were performed at a strain rate of 0.01/s using a 100 N load cell. Stress-strain curves were obtained from the raw data of load and displacement according to the tester. The elastic modulus was derived from the initial slope of the stress-strain curve after tensile testing. The tensile strength was calculated by dividing the maximum applied load to the cross-sectional area of the films. The elongation to failure was obtained from the stress strain curve at strain (%) where the specimen separated into half. Results were averaged on three independent measurements.

### 2.8. Clotting Assays

Two types of in vitro clotting assays were measured using an Organon Teknika Coag-A-Mate^®^XM (Durham, NC, USA) to perform activated partial thrombin time (APTT) assays. All surfaces were cleaned by typical household detergents and ethanol (75%) before each clotting experiment. In vitro clotting assays were performed at room temperature, and coagulation times were measured briefly as follows:

(1) 150 μL of plasma was added to a test tube along with 40 μL of CaCl_2_. The solution was mixed thoroughly and then placed onto surfaces of a glass plate (contact activation positive control) and PU samples to wait for 0, 7, 10, 14, 17, and 22 min (contact time). At each time interval, 100 μL of the plasma/CaCl_2_ sample was then placed into the Coag-A-Mate^®^XM and was immediately activated, using this step as a warming process, with a time of 180 s. An additional 100 μL of CaCl_2_ was added to the plasma/CaCl_2_ sample and clot formation was then recorded (clot time). Results were averaged on three independent measurements.

(2) 700 μL of plasma was added to test tubes containing the control (glass beads) or shredded square PU samples with approximately the same surface area. At predetermined time points, 100 μL of the plasma was taken out from the test tube and mixed with 40 μL of CaCl_2_ before placing into the Coag-A-Mate^®^XM for activation. An additional 100 μL of CaCl_2_ was added to the plasma/CaCl_2_ mixture and clot formation time was then recorded.

## 3. Results and Discussion

### 3.1. Appearance of the PU Films

**Figure 1:**
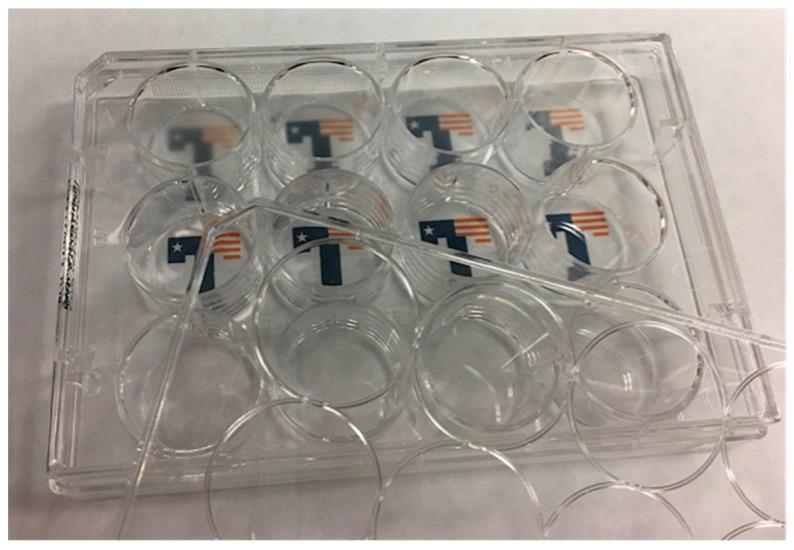
Physical appearance of the four polyurethane (PU) films: From left to right in the top wells, 200ET, 100ES, 60ES, and 60ET. The second row of four wells contains no PU films for comparison of the transparency of the PU films.

Four types of PU films, namely 60ES, 100ES, 60ET, and 200ET, were physically examined by eye. The films showed no differences in their appearance and texture and were free of defects, such as cracks and air bubbles. Thicker PU films (i.e., 100ES and 200ET) were found to be more opaque than the thinner counterparts (i.e., 60ES and 60ET) (Figure 1). During the study, PU films were stored in typical laboratory conditions (e.g., 23 ± 2 °C and 50 ± 5% RH) and showed no signs of degradation and/or changes in appearance.

### 3.2. FTIR and NMR Spectroscopy

FTIR spectroscopy is a nondestructive material characterization technique that can be used to quantitatively analyze the chemical structure of polymers. For example, FTIR analysis has been utilized to measure the physical state of pharmaceutical solids, including the amorphous/crystalline phases and the degree of crystallinity, based on the intensities of the characteristic peaks [22]. In addition, FTIR technique is very useful to study the urethane linkage (–NHCO–), as well as the composition of the soft segments in PU, such as ether (R–O–R’) or ester (R–COO–R’) [23,24]. Therefore, in this section, we explore the use of FTIR spectroscopy to determine the chemical structure of the various PU samples.

**Figure 2:**
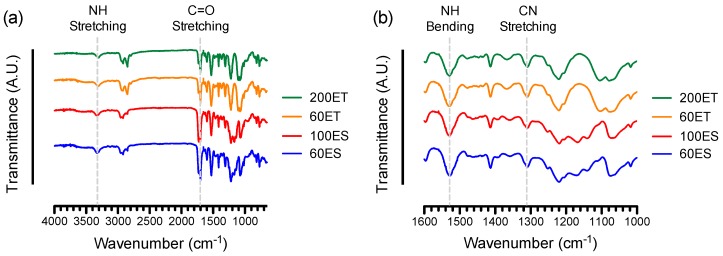
FTIR spectra of the various PU films for: (**a**) Wavenumbers from 650 to 4000 cm^−1^, showing the characteristic peaks of N–H stretching vibration at around 3300 cm^−1^ and C=O stretching vibration from amide I band at around 1700 cm^−1^; (**b**) wavenumbers from 1000 to 1600 cm^−1^, showing the characteristic peaks of N–H bending vibration from amide II band at around 1531 cm^−1^ and C–N stretching vibration from amide III band at around 1314 cm^−1^ that form the urethane linkage (–NHCO–).

The FTIR spectra of various PU samples showed a strong peak at 3330 cm^−1^, which is assigned to the stretching vibration of N–H groups in the urethane linkage (Figure 2a) [25]. This N–H peak from various PU samples was somewhat overlapping with the O–H stretching peak at 3400 cm^−1^, typically from the presence of moisture in the air or within the sample. Nonetheless, the O–H stretching peak was generally broader than the N–H peak, with less relative intensity as compared to other characteristic peaks in the 1000 to 1600 cm^−1^ region [26]. Furthermore, the frequency of N–H stretching vibration tended to shift toward 3400 cm^−1^, depending on the conformation of the urethane linkages with respect to one another under intermolecular hydrogen bonding. For example, the bands at 3325 cm^−1^ and 3313 cm^−1^ were associated with intramolecular hydrogen bonding of N–H and C=O within the urethane linkage, whereas the bands at 3440 cm^−1^ and 3451 cm^−1^ were attributed to the intermolecular hydrogen bonding [27]. Specifically, the intermolecular hydrogen bonds in PU formed at the carbonyl groups in urethane linkage of the hard segments and/or at the carbonyl groups (ester) or oxygen (ether) groups in the soft segments [28]. For poly(ester-urethane), the competition of hydrogen bonding between the carbonyl groups of the hard segments and the soft segments determined the molecular configurations [29]. The ability to form hydrogen bonds was critical to the surface polarity and hemocompatibility of the PU, as aliphatic PUs (all N–H groups are hydrogen bonded) tended to be more hydrophilic than aromatic PUs [30].

Further examination of the FTIR spectra from various PU samples suggests the characteristic peaks at 1700 cm^−1^, 1531 cm^−1^, and 1314 cm^−1^ were due to C=O stretching vibration (amide I band), N–H bending vibration (amide II band) and C–N (amide III band) stretching vibration, respectively (Figure 2b) [25]. In addition, the peak at 1597 cm^−1^ indicates the skeletal vibration of C=C in the aromatic ring [31]. The evidence of the aromatic ring conformation in various PU samples was supported by the coupling peak at 814 cm^−1^, the most significant characteristic peak for out of plane bending vibration of C–H in 1,4-disubstituted aromatic ring [32]. This finding suggests the chemical structure of hard segment in the various PU samples consisted of aromatic polymer, such as diisocyanates [33]. In contrast, the absorbance between 2930 and 2860 cm^−1^ (C–H stretching vibrations), coupled with the characteristic band patterns at 1200 and 1100 cm^−1^, were the characteristic absorptions of C–O–C stretching vibrations, indicating the composition of esters and ethers, respectively, in the soft segments of the various PU samples [31].

To further confirm the sequential structure of PU, ^1^H NMR at 400 MHz was recorded on the four types of PU films (Figures S1–S4). All samples show a small characteristic peak at 9.47 ppm, indicating the NH structure in urethane linkage [25]. Another important information depicted from the NMR spectra of all four PU samples include shifts at 7.31 and 7.04 ppm, which are assigned to the CH structure on the aromatic ring, located in the hard segments [25]. This finding supports the FTIR results of out of plane bending vibration of C–H in 1,4-disubstituted aromatic ring. Furthermore, noticeable differences in the NMR spectra of the ES and ET samples are found in the 1.58 ppm and 3.29 ppm shifts, which are among the largest peaks in the spectra. Specifically, the ES samples exhibit a smaller range of peaks between 1.49–1.67 ppm and a single peak at 3.30 ppm, whereas the ET samples show broader ranges of peaks between 1.46–1.67 ppm and 3.25–3.43 ppm. The differences on the ranges of these peaks suggest the CH shift in the soft segments of polyester and polyether, respectively [25]. In general, the NMR spectra show the characteristic peaks of various PU samples with information that are supportive of the FTIR study.

### 3.3. DSC

According to the FTIR spectra, the various PU samples consist of hard segments of diisocyanates and soft segments from esters and ethers, where the two segments are phase separated. DSC is a useful technique to quantify the phase transition temperatures of the two phases. In particular, the glass transition temperature of the soft segments occurs at around −45 to −50 °C, and this thermal event is used to measure the purity of the soft phase [34,35]. Due to the limit of the instrumentation, we performed DSC measurements on the various PU samples from 25 to 200 °C to focus on the thermal events above room temperature.

**Figure 3:**
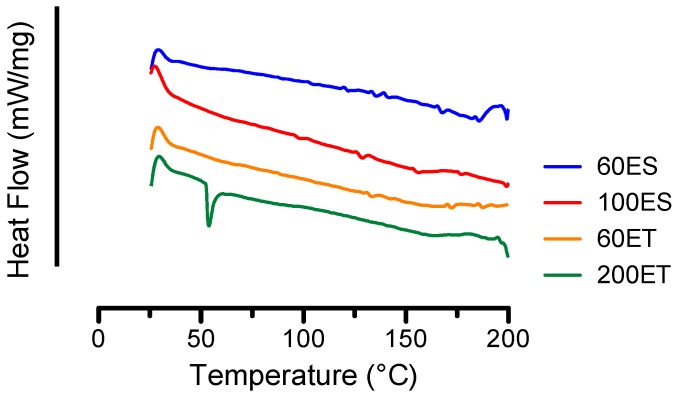
DSC thermograms of the various PU films from 25 to 200 °C, where a thermal endotherm at approximately 50 °C for the 200ET groups corresponds to melting of the soft segments.

The representative DSC thermograms from various PU samples are shown in Figure 3. As seen from the figure, 200ET samples exhibited a thermal endotherm at 55.1 ± 1.3 °C, corresponding to melting of the soft segments. This thermal event was not seen in the other PU samples, suggesting that the 200ET comprised a different degree of crystallinity in the soft segments than other the PU samples [36]. This finding is in agreement with the supplier’s manufacturing of PU samples where the 200ET samples were processed in a different batch.

As temperature increases, a small thermal endotherm was found at 124.1 ± 2.8 °C, 123.7 ± 3.2 °C, 119.8 ± 7.2 °C, and 126.7 ± 2.5 °C for 60ES, 100ES, 60ET, and 200ET, respectively. This thermal event is associated with the melting of hard segments that have long range ordering [35]. In particular, the 60ES and 100ES groups showed several other small thermal endotherms between 120 and 175 °C, suggesting that the poly(ester urethane) exhibited a mixing of crystalline regions with various range of ordering [37,38]. Finally, the thermal endotherms between 175 and 200 °C were associated with the melting of the microcrystalline hard segments. In general, the DSC thermograms informed the melting of the hard and soft segments and the crystallinity of the various PU samples. An increase in the crystallinity of the sample increases the temperatures of the corresponding thermal events.

### 3.4. TGA

**Figure 4:**
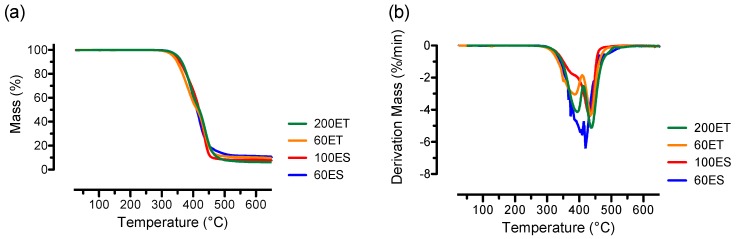
Thermal stability evaluations of the various PU films, showing (**a**) representative thermal gravimetric analysis (TGA) curves on percentage mass loss with onset temperatures ranged between 337 to 355 °C; and (**b**) representative dTG curves where the 60ES and 100ES groups exhibited single peak decomposition temperature and the 60ES and 200ES groups displayed a two-step decomposition process.

Thermal stability of the various PU films was investigated by using TGA, and the percentage mass loss of various PU films at temperatures from 25 to 650 °C is shown in Figure 4a. Our results show that the four types of PU films exhibited a similar mass loss profile with decomposition events that began at approximately 276 °C. Specifically, the average onset temperatures were 355.0 ± 2.1 °C, 359.3 ± 1.6 °C, 337.1 ± 2.0 °C, and 354.2 ± 2.1 °C for the 60ES, 100ES, 60ET, and 200ET groups, respectively. The mass loss associated with the onset temperature was attributed to the dissociation of urethane linkages, including diisocyanates, alcohol, and amines [39]. In addition to the onset temperatures, there were negligible mass losses (<0.1%) up to 250 °C for the various PU films, suggesting that these samples contained very minimal water content [40]. 

The derivative of the mass loss in TGA profiles yields dTG curves, and the dTG curves are useful in determining the peak decomposition temperatures, as well as the types of phase transitions during decomposition. As seen from the dTG curves shown in Figure 4b, both 60ES and 100ES samples exhibited a single peak located at an average temperature of 406.1 ± 13.9 °C and 433.3 ± 2.7 °C, respectively. In contrast, the 60ET and 200ET samples exhibited a two-stage decomposition process with the corresponding first peak located at an average temperature of 385.6 ± 5.0 °C and 394.1 ± 1.1 °C, and the corresponding second peak located at an average temperature of 437.8 ± 1.6 °C and 439.6 ± 1.7 °C, respectively. The peak decomposition temperatures with their corresponding percentage mass losses for 60ES, 100ES, 60ET, and 200ET are listed in Table 2.

### 3.5. XRD

While infrared techniques are useful in observing short-range molecular interactions, XRD provides information on the long-range ordering of the molecular chains. In addition, XRD method is particularly suited for the determination of crystallinity [41] and chain orientation [42] in the segmented PU. Furthermore, depending on the amount of hard and soft segments, phase separation may affect the structural regularity that leads to poor hemocompatibility.

**Figure 5:**
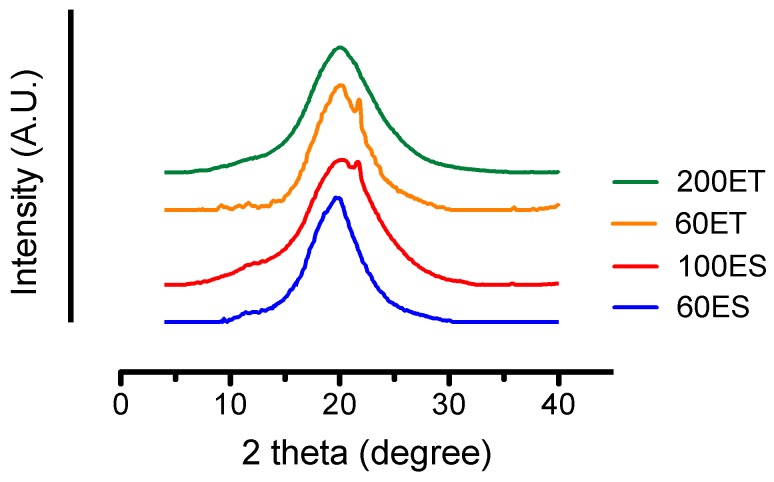
XRD patterns of the various PU films, showing a broad peak at 2θ = 20° related to semicrystalline structure of the hard/soft segments in PUs. The small peaks at the shoulders of the 100ES and 60ET groups are attributed to the partial crystallization of the soft segments.

The degree of crystallinity depends on the structure of diisocyanates, as they form the backbone of the PU. As seen from Figure 5, all of the PU films showed a broad peak at 2θ = 20°. The board peak, along with its small intensity, suggests that the PU samples were generally amorphous while carrying a small fraction of crystalline phase. The relatively low amount of the crystalline phase made the determination of the degree of crystallinity difficult. Furthermore, aliphatic PUs typically had a sharp peak due to the aligned chain orientation, while the aromatic PUs exhibited a much broader peak attributed to the amorphous structure [43]. Interestingly, the 60ET groups and the 100ES groups showed smaller peaks at 2θ = 21.8° indicating some level of partial crystallization of the soft segment [43]. Our finding in XRD is in agreement with the results from the FTIR analysis.

### 3.6. Water Content and Water Absorption

**Figure 6:**
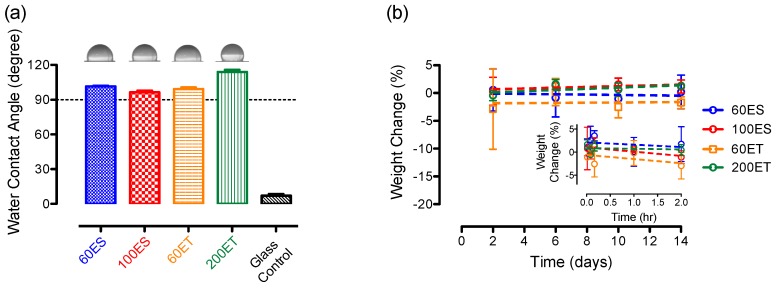
(**a**) Water contact angle study on the various PU films comparing to a glass control. (**b**) Water absorption and/or degradation study on various PU films over 14 days. Figure inset shows short-term study up to 2 h.

Surface hydrophilicity/hydrophobicity of a material is a critical factor that affects protein adhesion, and the surface properties are determined by the water contact angle experiment (Figure 6a). As seen from the representative image for each PU sample, the water droplet formed a sphere-like shape due to the balanced net force between surface hydrophobicity of the film and the surface tension of the water droplet. Our results indicate that the surfaces of various PU films were hydrophobic, with an average water contact angle of 101.5 ± 0.6, 96.4 ± 1.5, 99.2 ± 1.5, and 114.0 ± 1.8 for the 60ES, 100ES, 60ET, and 200ET groups, respectively. In comparison with a glass surface (control), which resulted in an average water contact angle of 7.0 ± 1.5, the significant high value of the contact angle from various PU films (*p* < 0.05) suggests that the PU samples were hydrophobic, perhaps due to the aromatic configuration in the molecular structure (Figure 2).

Water absorption determines the amount of water uptake in a material over a period of time. A higher water absorption rate may lead to a change in surface hydrophobicity, which can further alter the hemocompatibility. In the event of a mass loss, the information is valuable to give an early insight into polymer degradation or dissolution. According to the results, all four types of PU films exhibited a very minimal change in mass (< ±5%) over 14 days of observation (Figure 6b). In addition to the 14-day study, a short-term observation was performed to examine the water absorption behavior in the PU films (Figure 6b inset). Similar to the 14-day study, the various PU films showed a minimal mass change (< ±5%) over 2 h of incubation. Our results suggest that there was a minimal to negligible change on the mass of PU films, and this finding is supported by others who examined water absorption in PU films from 48 h to 6 months [44,45,46,47]. As a result, the various PU samples were stable under physiological condition without noticeable changes in hydrophobicity, as well as degradation behaviors.

### 3.7. Mechanical Properties

Studying the mechanical properties of PU films is crucial for the potential use of thromboresistant coatings on implantable devices. When introducing these devices (e.g., vascular stents) in the body, the shear force introduced by the blood flow may degrade coatings that have insufficient strength. Typical shear stresses ranged from 1.2 to 3 Pa for a laminar flow in vivo [48,49,50], and maximum shear stress occurs generally at 0.5-fold of the normal stress. More importantly, vascular stents are required to expand and attach to the blood vessel after they are inserted into the body. As such, coatings with proper elasticity are preferred for the use in thromboresistant application.

**Figure 7:**
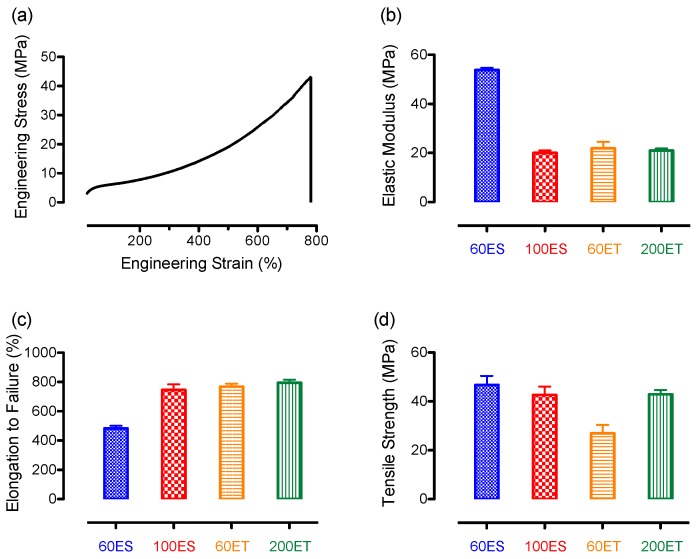
Mechanical properties of the various PU films, showing (**a**) a representative engineering stress-strain curve from 200ET groups; (**b**) average elastic moduli; (**c**) average elongation to failure; and (**d**) average tensile strength.

The mechanical properties of the TPU films were evaluated using tensile testing on an Instron materials tester with a force transducer under a constant strain rate in order to determine their mechanical properties. Stress-strain data obtained from the raw data during tensile testing were used to inform the chemical structure of the material [51]. A representative stress-strain curve for the 200ET film is shown in Figure 7a, and the stress-strain curves of all other PU films tested are similar to the representative curve. According to the stress-strain curve, an initial linear viscoelastic region can be seen where the increase of stress is proportional to the increase of strain. After the linear viscoelastic region, stress increases minimally with increasing strain (~400% strain). This region is associated with the unfolding of the soft segments. Since the PU samples are more aromatic than aliphatic, the unfolding of the soft segments produces large strain. After the unfolding region, a strain-hardening region can be found, where the significant increases of stress are accompanied with the increase of strain. This behavior is associated with the stretching of the molecular bonds that yields a much higher stress. Supported by the DSC data (Figure 3), the stress-strain curve shows that the PU samples exhibit a rubbery-like behavior with three distinctive regions [52].

Information on the elastic modulus can be obtained from the initial region, which is the slope at the beginning of the curve. The average moduli of the various PU films are shown in Figure 7b, and they are 53.8 ± 0.9 MPa, 20.0 ± 1.0 MPa, 21.9 ± 2.6 MPa, and 21.0 ± 0.9 MPa for the 60ES, 100ES, 60ET, and 200ET groups, respectively. The average modulus of the 60ES groups appears to be 2-fold higher than that of the other groups, suggesting that the material is intrinsically different than the others (e.g., crosslinking [53], hard/soft segment ratio [54], or types of hard/soft segments [55]).

Unfolding of the entangled molecular chain in the second region, primarily from the soft-segments, can be captured from the average elongation to failure. This property correlates to the overall percentage of deformation that was applied to the material before failure, and it is shown in Figure 7c. As seen from the figure, the average percentage elongation to failure was 482.4 ± 19.4%, 746.3 ± 37.9%, 768.7 ± 19.7%, and 794.9 ± 22.0% for the 60ES, 100ES, 60ET, and 200ET groups, respectively. These values match the findings from the elastic moduli, where the 60ES groups received approximately 0.6-fold decrease in average percent elongation to failure. This observation suggests that the 60ES groups are stiffer with less elasticity than other groups, suggesting the potential effects on crosslinking, different hard/soft segment ratios, or types of hard/soft segments used in the films.

The last region of the stress-strain curve is associated with strain-hardening effect, and this phenomenon is quantified by the tensile strength of the material, which occurs at the peak of the curve where the material fails. The average tensile strength of various PU films investigated is shown in Figure 7d, and they are 46.7 ± 3.7 MPa, 42.6 ± 3.4 MPa, 26.9 ± 3.4 MPa, and 42.9 ± 1.8 MPa for the 60ES, 100ES, 60ET, and 200ET groups, respectively. While the 60ES, 100ES, and 200ET groups receive very similar tensile strengths, the 60ET groups appear to be significantly weaker than the others (*p* < 0.05). In a study, the decrease of tensile strength was attributed to the steric hindrance of the increased concentration of the side dimethyl and methyl groups [56]. During constant strain, soft segment chains can be elongated and crystallized. This steric hindrance on the restriction of chain crystallization resulted in the decrease of the available sites for crosslinking and yielded a relatively low degree of microphase separation. This particular theory is further supported by the DSC data of 60ET and 200ET, where a melting of the soft segment was found in 200ET rather than the 60ET groups (Figure 3). Therefore, a decrease of tensile strength in the 60ET can be largely associated with this theory.

### 3.8. Clotting Assays

In light of the use of these PU samples as thromboresistant coatings on implantable devices, the compatibility of the various PU films with blood plasma needs to be determined. Several studies have shown that polyurethane is ideal for its thromboresistant activities [57,58,59]. Therefore, in this section, the hemocompatibility of each PU films is determined by evaluating the clotting rate against human blood plasma.

**Figure 8:**
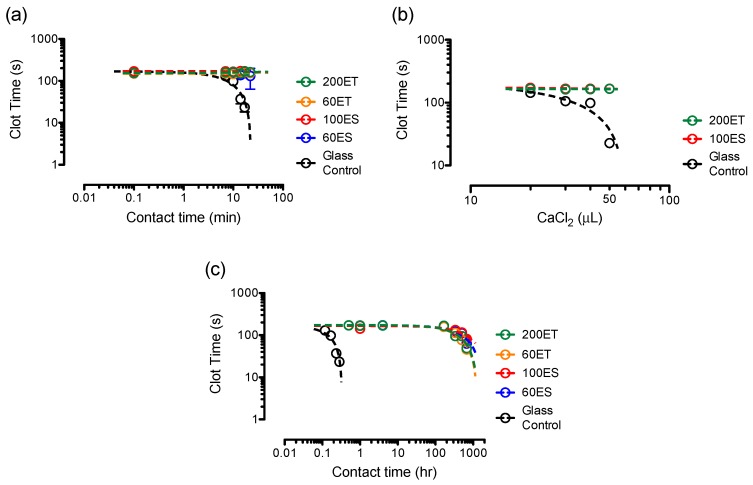
In vitro clotting assays on the various PU films in comparison with the glass control, showing (**a**) clotting assay (1) where human blood plasma were placed on the surface of the activating substances; (**b**) effects of CaCl_2_ concentrations on the clot times using clotting assay (1); and (**c**) clotting assay (2) where human blood plasma and the activating substances were placed in an enclosure.

To find the clot time, we initiated an in vitro clotting assay by placing a droplet of human blood plasma on the surfaces of the various PU films, in comparison with the surface of the glass control group (Figure 8a). Immediately after the contact with the PU surfaces (i.e., 0 min contact time), the clot times were 138 ± 16 s, 169 ± 4 s, 149 ± 3 s, and 154 ± 2 s for the 60ES, 100ES, 60ET, and 200ET groups, respectively. These clot times showed no statistical significance as compared to the glass control group (*p* > 0.05). At 10 min of contact time, the clot time for the glass control group was 98 ± 11 s, whereas the clot times for the various PU samples were 160 ± 17 s, 167 ± 2 s, 145 ± 19 s, and 163 ± 4 s for the 60ES, 100ES, 60ET, and 200ET groups, respectively. At the end of the assay (i.e., 22 min contact time), the plasma on the glass surface was completely clotted. In contrast, the clot times of various PU samples were 131 ± 67 s, 156 ± 6 s, 154 ± 20 s, and 162 ± 7 s for the 60ES, 100ES, 60ET, and 200ET groups, respectively. Our results indicate that the investigated PU films exhibited excellent hemocompatibility with almost no change in the clot time at various contact times up to 22 min.

In the first in vitro clotting assay, 40 μL of CaCl_2_ were added to the plasma to enable the clotting cascade by interacting with clotting factors, where the contact surface (e.g., various PU films or glass control) served as the activating substance [60]. As such, the concentration of the CaCl_2_ played an important role in determining the rate for clots to form. To observe the effects of CaCl_2_ on the clot time, we performed an additional in vitro clotting assay using 20–50 μL of CaCl_2_ on 100ES, 200ET, and glass control groups with 10 min of contact time (Figure 8b). Increasing calcium concentration from 20 to 50 μL decreased the clot times from 143 ± 3 s to 23 ± 1 s when the plasma were placed on the surface of glass control group. In contrast, the clot times for 100ES and 200ET groups were 172 ± 2 s and 167 ± 3 s at 20 μL of CaCl_2_ and were 167 ± 5 s and 166 ± 4 s at 50 μL of CaCl_2_, respectively. Our results suggest that calcium promoted the formation of clots, where the control group displayed a dependence on the calcium concentration and the PU groups showed an excellent hemocompatibility.

The first in vitro clotting assay was carried out in laboratory conditions where the plasma could dry out after 25 min. This assay limited the contact time to be no more than 22 min, and combining with the CaCl_2_ concentration assay with a maximum level of CaCl_2_ in the 50 to 60 μL range, clotting due to PU as an activating substance was unable to quantify. For this reason, we performed a second in vitro clotting assay where samples (plasma + activating substances) were placed into Eppendorf tubes that allowed for storage up to four weeks or longer. At pre-determined time intervals, a sample of plasma was taken out of the tube and placed into the coagulation analyzer to record the clot time (Figure 8c). Using this assay, the clot times of various PU groups showed a decrease of clot time with a contact time that was 1000-fold longer than the control glass surface. Specifically, the clot times were 63.3, 81.4, 45.8, and 48.9 s for the 60ES, 100ES, 60ET, and 200ET groups after 4 weeks of contact time (672 h), respectively. This finding is significant because, while the first in vitro clotting assay demonstrated the PU samples exhibited better hemocompatibility than the glass control, the length of contact time of the PU samples was determined in the second in vitro clotting assay.

Several studies have shown that modified PUs exhibit thromboresistant properties. For example, water-soluble chitosan/dextran sulfate was immobilized onto PU films and the clot time was 6.7-fold longer than the native PU after 30 min of contact time [61]. Others incorporated heparin into PU membranes to allow a slow release of heparin [62]. Results showed that heparin-loaded PU membranes exhibited clot times that were greater than 200 s after 30 days of contact time. More importantly, the control PU membranes showed a clot time of 46.8 s after the same contact time, and our findings are in agreement with the reported value. Furthermore, poly(ethylene glycol) monoacrylates (PEG-MAs) were grafted onto polycarbonateurethane films to increase the hydrophilicity of the films, which suppressed platelet adhesion to improve hemocompatibility [63]. In general, our results show that the investigated PU films exhibited excellent thromboresistant abilities that were ideal for the use of coating materials on artificial organs and/or implantable devices to minimize blood clotting.

## 4. Conclusions

In this work, we reported the thermal and physico-mechanical characterizations of various PU films for potential uses in thromboresistant coatings. The PU films, obtained from a supplier, included ester-based and ether-based soft segments with different thicknesses and shore hardness. In attempts of characterizations, we showed the differences in molecular configurations between the poly(ester urethane) films and the poly(ether urethane) films using FTIR. Thermal characterizations suggest a melting of the soft segments in 200ET groups, whereas poly(ether urethane) films exhibited a two-step decomposition process. XRD data revealed the partial crystalline structure of the PU films, which attributed to the excellent mechanical properties. The surfaces of the investigated PU films were hydrophobic with minimal water absorption and degradation behaviors over two weeks. Finally, in vitro APTT clotting assays confirmed the thromboresistant properties of the various PU films via contact pathway, which displayed a 1000-fold increase in contact time as compared to the control glass surface. In conclusion, our work demonstrates that PU films are suitable for thromboresistant coating on implantable medical devices, such as a vascular stent.

## Figures and Tables

**Table 1:** Polyurethane films used in this research work, including their abbreviations and manufacturing properties from the supplier.

Sample Abbreviation	Soft-Segment	Shore Hardness A	Thickness (μm)
60ES	Ester-based	92	60
100ES	Ester-based	85	100
60ET	Ether-based	92	60
200ET	Ether-based	85	200

**Table 2:** dTG temperatures and their corresponding percentage mass losses of the various PU films.

	First Step		Second Step	
	Temperature (°C)	Mass (%)	Temperature (°C)	Mass (%)
60ES	406.1 ± 13.9	52.2 ± 4.5	-	-
100ES	433.3 ± 2.7	33.2 ± 1.6	-	-
60ET	385.6 ± 5.0	68.1 ± 1.4	437.8 ± 1.6	32.2 ± 1.6
200ET	394.1 ± 1.1	69.0 ± 1.5	439.6 ± 1.7	34.3 ± 0.5

## Supplementary Materials

The following are available online at https://www.mdpi.com/2306-5354/6/3/69/s1, Figure S1: NMR spectrum of 60ES sample, Figure S2: NMR spectrum of 100ES sample, Figure S3: NMR spectrum of 60ET sample, Figure S4: NMR spectrum of 200ET sample.
