# Supplementary material for: Thermal and Physico-Mechanical Characterizations of Thromboresistant Polyurethane Films
Source: Bioengineering (Basel). 2019 Aug 14;6(3):69. doi: 10.3390/bioengineering6030069 (PMC6783839; doi:10.3390/bioengineering6030069)
Supplement: Supplementary file 1 [file bioengineering-06-00069-s001.pdf]

bioengineering

Thermal and Physico-Mechanical Characterizations of Thromboresistant Polyurethane Films

Aaron C. Wilson, Shih-Feng Chou, Roberto Lozano, Jonathan Y. Chen, and Pierre F. Neuenschwander

[1] Department of Mechanical Engineering, College of Engineering, The University of Texas at Tyler, 3900 University Blvd, Tyler, TX 75799, USA
[2] School of Human Ecology, College of Natural Sciences, The University of Texas at Austin, Austin, TX 78712, USA
[3] Department of Cellular and Molecular Biology, The University of Texas Health Science Center at Tyler, Tyler, TX 75708, USA
* Correspondence: schou@uttyler.edu; Tel: 1-(903)-566-6209

Received: 25 June 2019; Accepted: 7 August 2019; Published: date

Spplementary Materials

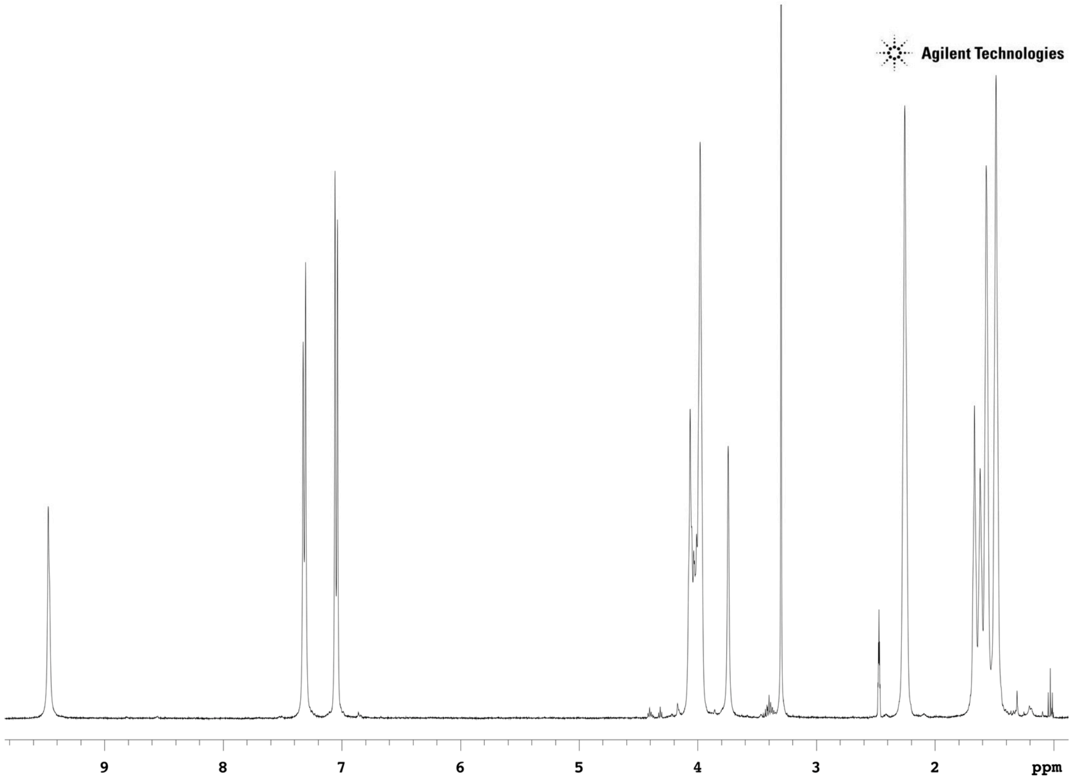

Figure S1. ^{1}H NMR spectrum of 60ES sample.

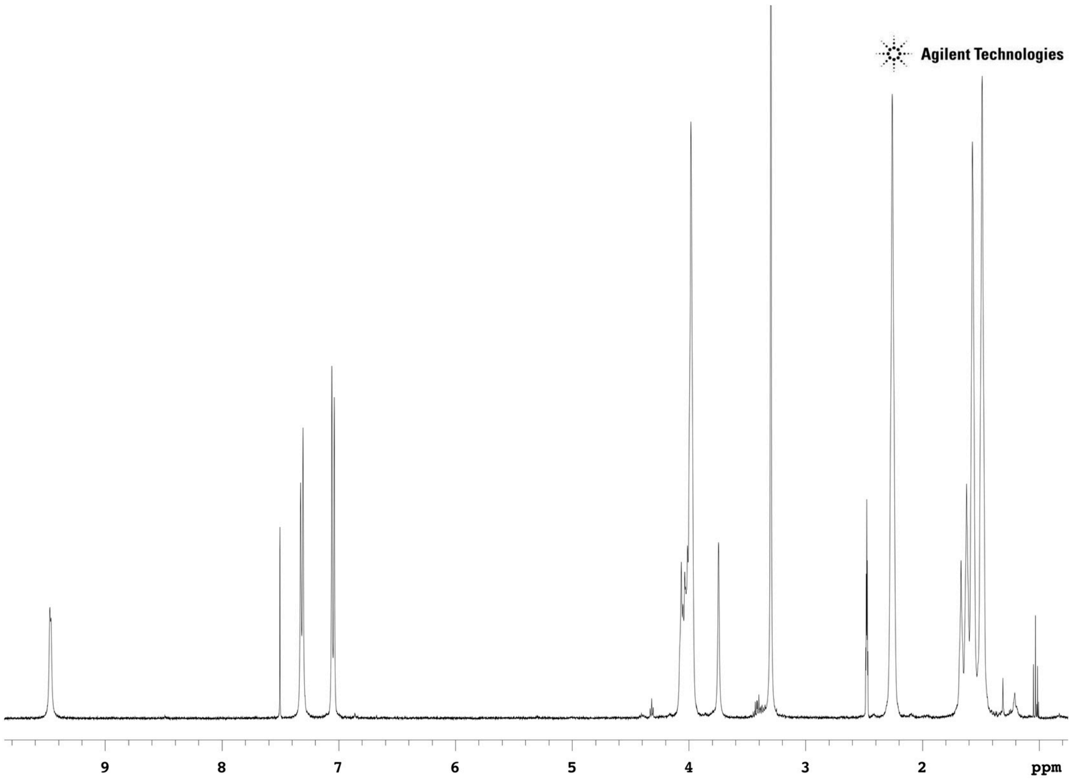

Figure S2. ^{1}H NMR spectrum of 100ES sample.

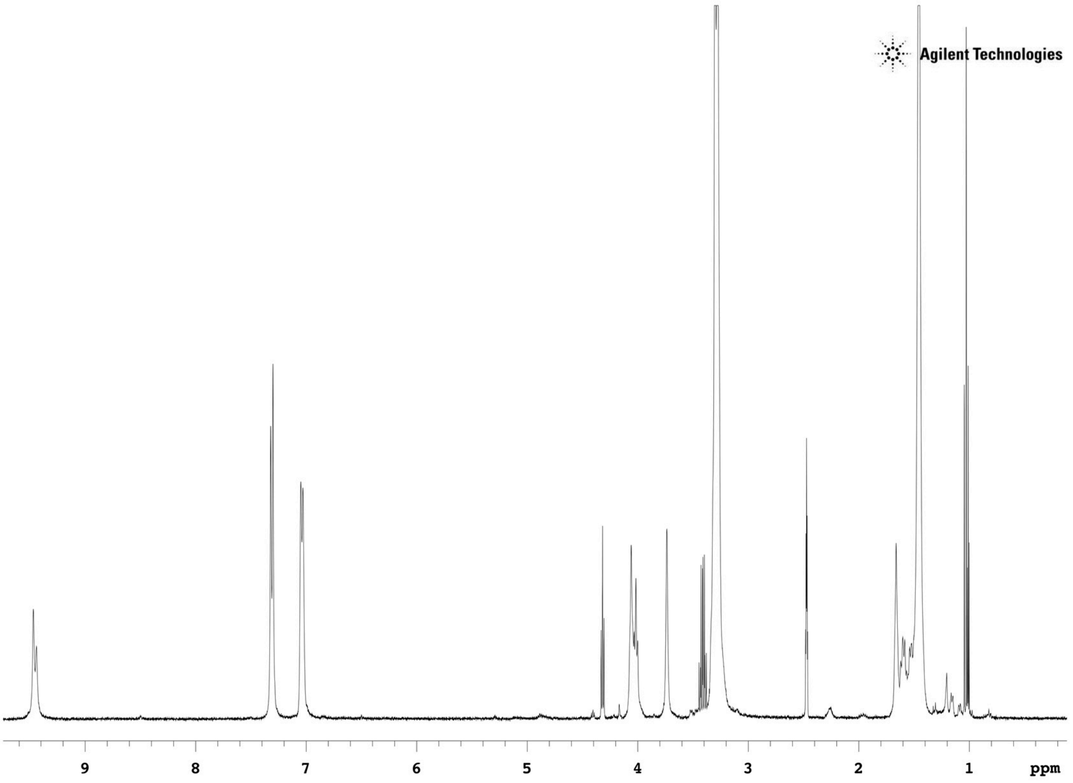

Figure S3. ^{1}H NMR spectrum of 60ET sample.

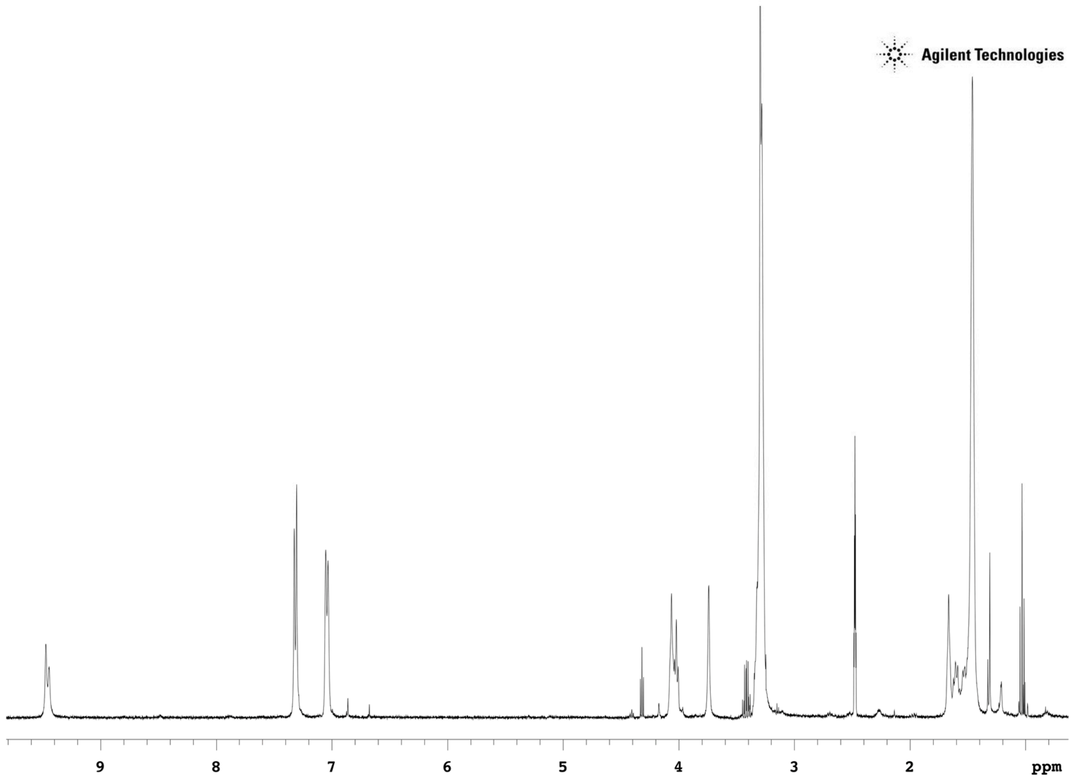

Figure S4. ^{1}H NMR spectrum of 200ET sample.
